# The application effect of mini midline catheters in intravenous therapy for neurology patients: real-world evidence from eastern China

**DOI:** 10.3389/fmed.2026.1836375

**Published:** 2026-06-23

**Authors:** Xiu Ding, Rongrong Mao, Tianyu Ma, Xiaoyan Li, Yanli Chen

**Affiliations:** Neurology Department, The First Affiliated Hospital of Soochow University, Suzhou City, Jiangsu Province, China

**Keywords:** mini midline catheter, intravenous therapy, ultrasound guidance, neurology department, complications

## Abstract

**Aim:**

To investigate the application effect of ultrasound-guided combined with Accelerated Seldinger Technique (AST) for mini midline catheter placement in patients in the neurology department undergoing short- to mid-term intravenous therapy, and to analyze its safety, feasibility, and influencing factors.

**Methods:**

A convenience sampling method was used to select 136 patients admitted to the Neurology Department of the First Affiliated Hospital of Soochow University from May to November 2025, who underwent mini midline catheter placement. Patient general information, intravenous therapy details, catheterization operation indicators, catheter indwelling outcomes, and complication occurrences were recorded. Statistical analysis was performed using SPSS 27.0.

**Results:**

All 136 patients were successfully catheterized. The one-time puncture success rate was 77.2%, the mean operation time was (9.15 ± 4.11) min, and the pain score during placement was low (1.74 ± 1.08 points). The mean catheter indwelling time was (12.44 ± 6.66) days. The overall complication rate was 11.0%, with catheter blockage being the most common (5.1%). A higher daily average infusion volume was associated with a higher incidence of complications (*P* < 0.05) and with a longer indwelling time (r = 0.351, *P* < 0.001). Patient satisfaction reached 89.7%.

**Conclusion:**

The ultrasound-guided mini midline catheter is safe and feasible for application in neurology department patients. It offers advantages including a high success rate of catheterization, low complication rate, and high patient satisfaction, making it particularly suitable for short- to mid-term intravenous therapy with a relatively high daily infusion volume. Future multicenter, large-sample studies are needed to further validate its clinical benefits.

## 1 Introduction

The neurology department mainly treats elderly patients with critical conditions such as cerebrovascular diseases, neurological degenerative diseases, and central nervous system infections. These patients often have underlying diseases that can lead to poor vascular elasticity and high fragility, such as hypertension, diabetes, and arteriosclerosis. Treatment usually requires continuous infusion of hypertonic and irritating drugs, such as mannitol, high-concentration electrolytes, and some antibiotics, as well as frequent venous blood sampling. The traditional repeated peripheral venous puncture not only increases patient discomfort, brings higher risks of phlebitis, unplanned extubation, and drug extravasation, but also increases the workload of nurses, seriously affecting the treatment comfort and continuity of patients (1–3). Central venous catheters (CVC) and peripherally inserted central catheters (PICC) can solve some problems, but they have more serious issues such as large trauma, high cost, and related complications risks, such as infection and thrombosis (4). The mini midline catheter, as an emerging venous access device, is a peripheral venous access device between short cannulas and medium-long catheters, with a length of approximately 8–10 cm, a retention time generally of 1–4 weeks, and a tip position not exceeding the level of the armpit (5, 6). The mini-midline catheters of the two specifications used in this study are intended for a maximum indwelling period of 28 days. It can avoid the serious complications of CVC and PICC, effectively protect peripheral blood vessels, reduce the pain caused by repeated punctures for patients, and has a high success rate of catheterization, low invasiveness, and low catheterization and maintenance costs (4, 7–9). Currently, there are more extensive studies on mini midline catheters abroad, mostly concentrated in emergency departments, intensive care units, or other urgent and critical departments where vascular access establishment is difficult (5, 9–11, 15). However, in China, the application and research of mini midline catheters are at the initial stage, mainly concentrated in surgery, oncology, or emergency departments (16–18). The application research for this specific patient group in neurology is still insufficient. Its applicability, safety, retention effect, and patient experience in this patient group still need high-quality localized research evidence to support. Since May 2025, our department has carried out ultrasound-guided combined accelerated Seldinger technique (AST) mini midline catheter insertion technology in neurology patients, successfully catheterizing 136 patients and achieving good results. The following is the report.

## 2 Methods

### 2.1 Study design

This was a prospective, observational, single-center study conducted from May to November 2025 in the neurology department of a tertiary grade A general hospital in Suzhou City, targeting patients who received intravenous infusion therapy with mini midline catheters inserted.

### 2.2 Participants and sample size

Patients admitted to the neurology ward of a tertiary-level Class A hospital in Suzhou between May and November 2025 who underwent placement of a mini midline catheter were selected as study subjects using convenience sampling. Inclusion criteria include: Age ≥ 18 years; Expected intravenous therapy duration of 1–4 weeks; Alert and able to cooperate with the study; Signed informed consent; Placement of a mini midline catheter for intravenous infusion therapy; Mini-midline catheter indwelling duration ≥ 24 h. Exclusion criteria include: Active infection, deformity, or severe skin disease at the catheter insertion site; Coagulation disorders; Allergy to mini midline catheter materials; Prolonged transfer out of the neurology ward during mini midline catheter placement; Concurrent placement of other central venous catheters where complications cannot be distinguished; Concurrent participation in other interventional studies.

According to Kendall's sample size estimation method, the minimum sample size is 5–10 times the number of items (12). Considering a 20% attrition rate, the sample size formula is calculated as follows: Number of items × (5–10) × (1 + 20%). This study includes 12 entries. Calculated using the formula 12 × (5–10) × (1 + 20%), the minimum sample size ranges from 72 to 144.

### 2.3 Pipeline retention and maintenance

The nurses who performed the ultrasound-guided catheter placement in this study participated in the “Central Line Insertion and Maintenance” course in 2025. This course was accredited by the Infusion Nurses Society (INS) and carefully designed in accordance with standards of practice for infusion therapy; they successfully obtained certificates of completion. Currently, our department has a total of 25 nurses, four of whom are qualified to perform this catheterization procedure. Prior to catheterization, the operator comprehensively assessed the patient's condition, treatment plan, and upper limb venous status, and obtained signed informed consent. The patient was positioned supine or in a semi-recumbent position with the upper limb on the puncture side abducted. A high-frequency linear array ultrasound probe was used to visualize upper arm vessels, clearly distinguishing arteries and veins in cross-section. The basilic vein was the primary choice, preferred for its straight course and suitable diameter, with the brachial vein, and cephalic vein serving as the secondary option. The placement of mini midline catheters is a new nursing technique introduced in our department. Since we had no prior experience to draw upon, we sought assistance from radiologists and reviewed relevant literature, which indicated that a ratio of 30% to 45% is optimal (13, 14). Based on the internal diameter of the target vein as measured by ultrasound, we strictly adhered to the principle that the “catheter-to-vessel diameter ratio should be ≤ 45%.” The ultrasound equipment used for catheterization in this study was a Mindray UMT-400. The ultrasound probe model was L12-3RCs. The catheter brand used in this study was PowerMe, available in two sizes: 20G × 10 cm and 22G × 10 cm. Under ultrasound guidance, identify and mark the optimal puncture site at least 5 cm above the elbow crease. The operator performs surgical hand antisepsis and establishes a maximized sterile barrier. During catheterization, we utilized a disposable central venous catheterization kit designed for the placement of peripherally inserted central catheters (PICC) or single-use central venous catheters (CVC), as well as for puncture and dressing changes. The sterile barrier supplies listed in the product's accessory list included surgical gloves, drapes (100 cm × 100 cm), and treatment sheets (50 cm × 60 cm). Centered on the puncture site, mechanically rub the area three times sequentially with 75% alcohol and 2% chlorhexidine gluconate ethanol solution (or povidone-iodine with an effective iodine concentration ≥0.5%), covering a diameter ≥20 cm. Allow the area to air dry. Under real-time ultrasound guidance, the operator stabilizes the transducer with the left hand to center the target vein on the screen. Holding the needle like a pen in the right hand, the operator inserts the needle at a 30°-45° angle approximately 0.5 cm lateral to the transducer. Closely monitor the screen to track the needle tip until it enters the vascular lumen and blood return is observed. Subsequently, reduce the angle and advance the needle slightly in parallel. After confirming unobstructed blood return, gently advance the guidewire. Adjust direction upon encountering resistance rather than forcing advancement. Once the guidewire is in place, advance the catheter along it at a constant speed to the predetermined length, then withdraw the needle core and guidewire. Re-scan using longitudinal ultrasound to confirm the entire catheter length is within the vascular lumen and the tip has not crossed the axillary level. During the procedure, the probe can be oriented perpendicular to the target vessel (short-axis view) or parallel to the target vessel (long-axis view), depending on the operator's preference. In this study, we first used the short-axis view for positioning and puncture; after confirming backflow, we rotated the probe to the long-axis view to verify that the catheter was within the vascular lumen. Upon observing blood during withdrawal, immediately flush the catheter with 10 ml of saline using a “push-pause-push” pulsatile technique, followed by positive pressure sealing. Finally, secure the catheter tension-free using a dedicated fixation device and sterile transparent dressing, clearly labeling all placement information. The catheter does not need to be sutured to the skin for fixation; we simply use a transparent dressing (film-coated, 10 cm × 12 cm, square).

Maintenance during the placement of mini midline catheters is critical to ensuring their safety and functionality. The responsible nurse must assess the puncture site daily, observing for redness, swelling, bleeding, exudate, pain, or catheter displacement. Transparent dressings should be changed every 7 days, or immediately if contaminated, damp, curled, or loose. Infusion connectors should be routinely replaced every 7 days in conjunction with dressing changes. Strictly adhere to proper flushing and capping procedures: Prior to each infusion, perform a 10 mL saline pulse flush followed by aspiration to verify patency. After administering hypertonic or viscous solutions such as mannitol, parenteral nutrition, or blood products, immediately perform a 20 mL saline pulse flush to reduce catheter clogging risk. During treatment intervals, routinely perform positive-pressure flushing with 10 mL saline every 12 h to seal the line. Simultaneously, closely monitor patients for unexplained fever or swelling/pain in the catheter-insertion site limb, remaining vigilant for catheter-related complications. All assessments, maintenance procedures, and patient complaints are fully documented in a standardized catheter observation log to ensure continuous quality monitoring.

### 2.4 Measurement

The demographic information section comprises eleven items to collect participants' demographic details: gender, age, educational attainment, disease diagnosis, number of prior chronic conditions, total hospitalizations, height, weight, BMI, upper arm circumference on the catheter side, and upper arm skinfold thickness.

Information related to intravenous therapy includes participants' average daily infusion frequency, average daily infusion volume, and classification and statistical analysis of various intravenous medications used. This encompasses isotonic/near-isotonic solutions (e.g., 0.9% sodium chloride injection, 5% glucose injection), hypertonic solutions (e.g., mannitol, glycerol fructose, 50% glucose injection), strongly irritant agents (e.g., sodium valproate injection), vasoactive agents (e.g., norepinephrine, urapidil hydrochloride injection, nicardipine hydrochloride injection), parenteral nutrition solutions (e.g., TNA), and blood products (e.g., plasma, human albumin, immunoglobulin).

Indicators for mini midline catheters include catheterization procedure metrics, catheter outcomes, and complication types. Catheterization procedure metrics include the vein punctured (e.g., basilic vein, brachial vein, cephalic vein and superficial veins of the forearm), target vessel diameter, number of puncture attempts, procedure duration (the procedure duration is measured from the moment the catheterization kit is opened until the catheter is successfully inserted and properly secured, measured in minutes), and pain score during catheterization (using the Numerical Rating Scale with scores ranging from 0 to 10, where “0” indicates no pain and “10” indicates the most severe pain). Catheter outcomes include indwelling duration (total days the catheter remains in the blood vessel, calculated from the day of placement to the day of removal), and reasons for removal (e.g., completion of therapy, occurrence of complications, unplanned removal, patient request for removal, transfer to another department or discharge with catheter in place, patient death, etc.). Document occurrence of complications and classify them specifically, including: puncture site bleeding/drainage, catheter occlusion, phlebitis (graded per 2021 INS criteria), catheter-related thrombosis (ultrasound diagnosis), and catheter-related bloodstream infection. Patient satisfaction (surveyed at extubation or prior to participant discharge using a 3-point Likert scale. Participants select “dissatisfied,” “neutral,” or “satisfied”).

### 2.5 Ethical considerations

This study has been approved by our hospital's Ethics Committee (Approval No. (2025) Ethics Approval No. 580). During the study, all patients or their family members signed informed consent forms for catheter placement. All patients' personally identifiable information (such as names, hospital ID numbers, etc.) was replaced with anonymous codes during the data collection phase to ensure data cannot be traced back to specific individuals, thereby maximizing the protection of patient privacy.

### 2.6 Data collection

Data collection took place between May and December 2025. Prior to catheter placement, researchers explained the necessity of the procedure, potential catheter-related risks, and the study objectives to participants, obtaining their informed consent. Following catheter placement, the catheter placement operator collected relevant procedural metrics, with a catheter placement assistant verifying the accuracy of these metrics. Following catheter insertion and removal, nurses proficient in micro-midline catheter procedures collected patient demographics, intravenous infusion regimens, catheter retention outcome measures, and complication rates through face-to-face interviews with participants and by retrieving data from HAITAI (an electronic health app used by physicians within the hospital) and Mobile Nursing (an electronic health app used by nurses within the hospital). Departmental head nurses ultimately cross-verified all data to ensure accuracy.

### 2.7 Bias control

A standardized protocol was provided to all researchers, clearly defining the study objectives, survey content, inclusion and exclusion criteria, and logical control design. Collected data underwent double-entry verification by two researchers. For responses exhibiting logical inconsistencies or raising questions during verification, researchers traced back to the study participants to determine the final correct answer.

### 2.8 Data analysis

Data processing and analysis were performed using SPSS 27.0 software. For continuous variables, those meeting normal distribution were expressed as mean ± standard deviation (x ± s), while those not meeting normal distribution were presented as median (interquartile range). Categorical variables were described using frequency and percentage (*n* (%)). For intergroup comparisons, normally distributed quantitative data with equal variances were analyzed using independent samples *t*-tests, while multiple group comparisons employed one-way analysis of variance (ANOVA). Categorical data comparisons utilized chi-square tests or Fisher's exact probability test. Pearson correlation analysis explored linear relationships between continuous variables. A *p*-value < 0.05 was considered statistically significant. Due to the limited total number of complication events (*n* = 15), a multivariate regression analysis was not feasible as it would violate the recommended events-per-variable criterion (at least 10 events per predictor variable). Therefore, univariate analyses were used to identify potential risk factors, and the results are interpreted as hypothesis-generating. All results are presented in three-line tables.

## 3 Results

### 3.1 Patient demographics and intravenous therapy status

This study included 136 patients in the neurology department who underwent placement of a mini midline catheter. Among them, 79 were male (58.1%) and 57 were female (41.9%); the mean age was (68.24 ± 13.70) years. The primary diagnosis was cerebral infarction (78 cases, 57.4%). The most common daily infusion frequency was twice daily (54 cases, 39.7%), and the most frequent daily infusion volume range was 500–1000 ml (77 cases, 56.6%). Regarding drug administration, all patients (136 cases, 100.0%) received isotonic or near-isotonic solutions; highly irritant solutions were used in 87.5% (119 cases), while hypertonic solutions were administered in 33.8% (46 cases). Vasoactive agents and blood products were used in 11.0% (15 cases) and 12.5% (17 cases), respectively. See Tables 1, 2.

**Table 1:** General patient information and baseline characteristics (*n* = 136).

Characteristics	Value
Sex [*n*(%)]	
Man	79 (58.1)
Female	57 (41.9)
Age (year)	68.24 ± 13.70 (31–91) ^a^
Education level [*n*(%)]	
Illiterate	18 (13.2)
Primary school	37 (27.2)
High school	55 (40.4)
Bachelor's degree or higher	26 (19.1)
Diagnosis [*n*(%)]	
Cerebral infarction	78 (57.4)
Cerebral hemorrhage	9 (6.6)
Epilepsy	2 (1.5)
Transient ischemic attack	2 (1.5)
Cerebral artery stenosis	7 (5.1)
Parkinson's syndrome	4 (2.9)
Others	34 (25.0)
Number of chronic diseases [*n* (%)]	
0	21 (15.4)
1	47 (34.6)
2	49 (36.0)
3	18 (13.2)
4	1 (0.7)
Number of hospitalizations [*n* (%)]	
1	69 (50.7)
2	30 (22.1)
3	14 (10.3)
4 or more	23 (16.9)
BMI(kg/m^2^)	23.52 ± 4.03 (15.6–40.1) ^a^
Upper arm circumference (cm)	26.59 ± 3.78 (16.0–37.0) ^a^
Subcutaneous fat thickness of the upper arm (mm)	1.58 ± 0.60 (0.5–3.8) ^a^
Diameter of the vein being punctured (mm)	3.39 ± 1.67 (1.0–15.0) ^a^

a, Data are presented as “mean ± standard deviation (range).”

**Table 2:** Intravenous therapy and drug use of patients (*n* = 136).

Items	*n*	Percent (%)
Daily average frequency of intravenous infusion		
1	24	17.6
2	54	39.7
3	41	30.1
4	7	5.1
5	8	5.9
6	2	1.5
Daily average infusion volume (ml)		
< 500	10	7.4
500–1000	77	56.6
1000–1500	35	25.7
≥1500	14	10.3
Medication use		
Isotonic/nearly isotonic drugs	136	100.0
Hyperosmolar drugs	46	33.8
Strongly irritating drugs	119	87.5
Vasodilators	15	11.0
Parenteral nutrition solution	5	3.7
Blood products	17	12.5

### 3.2 Catheterization procedure outcomes and catheter-related outcomes

All 136 patients successfully received mini-midline catheter placement. The catheterization procedures and indwelling outcomes are as follows. The basilic vein was the primary puncture site (105 cases, 77.2%). Single-puncture success was achieved in 105 cases (77.2%). The average procedure duration was (9.15 ± 4.11) minutes, with low catheter placement pain scores [(1.74 ± 1.08) points]. The mean catheter indwelling duration was (12.44 ± 6.66) days. The primary reason for catheter removal was successful completion of treatment (111 cases, 81.6%), while 16 cases (11.8%) required removal due to complications. The overall complication rate during indwelling was 11.0% (15/136), including catheter occlusion in 7 cases (5.1%), drug extravasation in 4 cases (2.9%), bleeding at the puncture site in 2 cases (1.5%), and one case each of phlebitis and catheter-related thrombosis (0.7%). Patient satisfaction was high, with 122 cases (89.7%) reporting satisfaction, as shown in Table 3.

**Table 3:** Catheterization operation and catheter indwelling outcomes (*n* = 136).

Items	Value
Vein being punctured [*n* (%)]	
Basilic vein	105 (77.2)
Cephalic vein	21 (15.4)
Brachial vein	10 (7.4)
Number of punctures [*n* (%)]	
1	105 (77.2)
2	19 (14.0)
3 or more	12 (8.8)
Duration of catheter insertion procedure (min)	9.15 ± 4.11 (2–30)^a^
Pain score for catheter insertion ^b^	1.74 ± 1.08 (1–5)^a^
Duration of catheter insertion	12.44 ± 6.66 (2–32)^a^
Reason for extubation [*n* (%)]	
Treatment completed ^c^	112 (82.3)
Complications	16 (11.8)
Unplanned removal ^d^	2 (1.5)
Retained catheter	6 (4.4)
Number of complications [*n* (%)]	15 (11.0)
Type of complications [*n* (%)]^e^	
Phlebitis	1 (0.7)
Extravasation of the medicinal liquid	4 (2.9)
Excessive bleeding at the puncture site	2 (1.5)
Catheter blockage	7 (5.1)
Catheter-related thrombosis	1 (0.7)
Patient satisfaction [*n* (%)]	
Not satisfied	1 (0.7)
Generally	13 (9.6)
Satisfied	122 (89.7)

^a^, Data are presented as “mean ± standard deviation (range).” ^b^, Pain scores are measured using the Numerical Rating Scale (NRS), with a range of 0–10. ^c^, “End of treatment” refers to planned catheter removal, indicating that the catheter has fulfilled its intended therapeutic purpose. ^d^, “Unplanned catheter removal” refers to unexpected removal not caused by the end of treatment or complications (e.g., catheter dislodgement). ^e^, Complications are counted as individual events; multiple complications occurring in the same patient are recorded separately.

### 3.3 Factors associated with complications

Univariate analysis revealed that the average daily infusion volume in patients with complications was significantly higher than in those without complications, with the difference being statistically significant (*P* < 0.05). Due to the limited number of complication events, multivariate analysis was not conducted; thus, infusion volume should be considered a potential risk factor rather than an independent predictor. This study also analyzed continuous variables such as age, body mass index, vein diameter, and puncture duration, as well as categorical variables including the use of hypertonic solutions, highly irritating agents, and vasoactive drugs. Results indicated no statistically significant differences or associations between these factors and the occurrence of complications (*P* > 0.05), as shown in Table 4.

**Table 4:** Univariate analysis of complications related factors (*n* = 136).

Items	Non-complication group (*n* = 121)	Complication group (*n* = 15)	*t/χ^2^*	*P-value*
Age (years)	67.83 ± 13.78	71.53 ± 12.99	*t* = −0.989	0.325
BMI (kg/m^2^)	23.51 ± 3.82	23.65 ± 5.61	*t* = −0.126	0.900
Diameter of the vein (mm)	3.41 ± 1.69	3.23 ± 1.60	*t* = 0.392	0.696
Infusion volume (ml)	898.84 ± 447.64	1174.67 ± 729.42	*t* = −2.078	**0.040**
Duration of catheter insertion procedure (min)	9.21 ± 4.13	8.67 ± 4.05	*t* = 0.479	0.633
Hyperosmolar drugs	40(33.1)	6(40.0)	*χ^2^ =* 0.287	0.592
Strongly irritating drugs	106 (87.6)	13(86.7)	*χ^2^ =* 0.011	0.918
Vasodilators	12 (9.9)	3(20.0)	*χ^2^ =* 1.383	0.24

Bold values indicate statistical significance (*P* < 0.05).

### 3.4 Factors influencing catheter retention duration

This study employed one-way analysis of variance and Pearson correlation analysis to investigate factors influencing catheterization duration. Results demonstrated statistically significant differences in catheterization duration among patients with different reasons for catheter removal (*P* < 0.05). Furthermore, catheterization duration showed a significant positive correlation with the average daily infusion volume (r = 0.351, *P* < 0.001). However, the choice of puncture vein, patient age, and target vein diameter were not significantly associated with catheterization duration (*P* > 0.05), as shown in Table 5.

**Table 5:** Analysis of factors related to the indwelling time of mini midline catheters (*n* = 136).

Items	*Test statistic (F or r)*	*P-value*
Vein being punctured	*F = 0.708*	0.494
Reason for removal	*F = 3.596*	**0.008**
Age	*r = 0.002*	0.985
Diameter of the vein	*r = 0.092*	0.288
Infusion volume	*r = 0.351*	**< 0.001**

F values are from one-way ANOVA for categorical variables. r values are Pearson correlation coefficients for continuous variables. Bold values indicate statistical significance (*P* < 0.05).

## 4 Discussion

### 4.1 Safety and feasibility of mini-midline catheters in neurological applications

The results of this study indicate that ultrasound-guided mini-midline catheters represent a safe, feasible, and viable vascular access option for neurology patients, capable of meeting their short-to-medium-term treatment needs. Despite the complex composition of intravenous medications administered to neurology patients—including a high proportion of potent stimulants (87.5%) and hypertonic solutions (33.8%), along with vasoactive agents, parenteral nutrition solutions, and blood products—the catheters demonstrated overall favorable retention rates with a low complication incidence. These findings are comparable to those reported in a study involving oncology patients (18). This seemingly paradoxical outcome hinges on the choice of puncture site: all catheters were placed under ultrasound guidance into the deep veins of the upper arm. Positioning the catheter tip within the blood-rich deep veins of the upper arm allows rapid dilution of medications, significantly reducing the risk of phlebitis caused by the hypertonic and highly irritating agents commonly used in neurology. Simultaneously, ultrasound guidance ensures precise puncture, avoiding vascular damage from repeated attempts (19). The single-attempt catheterization success rate in this study was 77.2%, comparable to results reported in acute pancreatitis patients (16). This suggests that the core technical advantages of this approach may help maintain consistent catheterization efficiency across different clinical settings (20). Notably, the mean age of neurology patients in this study was 68.24 years, potentially higher than emergency populations, indicating more challenging vascular conditions. Achieving comparable catheterization success rates under these circumstances further validates the technique's applicability for patients with poor vascular access. This advantage directly translates to favorable clinical outcomes: the mean catheter indwelling time reached (12.44 ± 6.66) days, meeting the treatment duration requirements for the vast majority of patients; The overall complication rate was low (11.0%), primarily consisting of minor events such as catheter occlusion. Ultimately, patient satisfaction reached 89.7%, a finding comparable to a randomized controlled trial from Italy involving emergency medical and surgical patients (21). Consequently, for neurology patients requiring infusion of multiple irritant medications, the mini midline catheter may offer a reliable alternative compared to traditional peripheral indwelling needles in this specific patient population, although confirmatory comparative studies are needed.

### 4.2 Risk factor analysis and management implications for complications

This study found that the overall complication rate for mini midline catheters was relatively low (11.0%), with complications primarily including catheter occlusion, drug extravasation, puncture site bleeding, and phlebitis. These findings are consistent with those of a foreign study (21). In-depth analysis revealed that daily infusion volume was the only risk factor significantly associated with complication occurrence (*P* < 0.05). Patients who developed complications had a significantly higher daily infusion volume [(1174.67 ± 729.42) ml] compared to those without complications [(898.84 ± 447.64) ml]. In this study, the use of hypertonic solutions and highly irritating agents—such as mannitol, glycerol fructose injection, edaravone injection, and gangliosides—did not independently demonstrate a significant association with complication rates. This may be attributed to the placement of all catheters in the high-flow deep veins of the upper arm, where rapid blood flow dilution partially counteracted the physicochemical irritation of these medications. Notably, catheter occlusion was the most common complication (5.1%), exceeding rates reported in tumor patients (18). This may relate to reduced limb mobility and slower blood flow in neurology patients, particularly post-stroke individuals, suggesting enhanced emphasis on prophylactic flushing and early mobilization guidance for this population.

Based on the above analysis, we propose the following management insights: First, patients with high daily infusion volumes (>1000 ml) should be considered high-risk for complications. Clinicians should increase monitoring frequency of the catheter insertion site and the limb, strictly adhere to pulsatile flushing and positive pressure occlusion protocols, and consider shortening flushing intervals. Second, although deep upper arm venous catheterization provides a relatively safe foundation for administering irritant medications, it is still essential to adhere to drug labels and guideline recommendations, avoiding continuous infusion of foaming agents or medications with extreme pH values through this catheter. Finally, given the reduced mobility of neurology patients, nursing staff should actively encourage and guide passive or active limb exercises on the catheterized side whenever clinically feasible. This promotes venous return and serves as a crucial non-pharmacological measure for preventing catheter-related thrombosis and occlusion.

### 4.3 Factors influencing retention duration and their clinical significance

This study found that catheter dwell time is primarily influenced by treatment requirements and outcomes, suggesting that this catheter can effectively adapt to and support clinical treatment cycles of varying intensity. Effective catheter dwell time serves as a key indicator for evaluating its clinical utility. Results indicate that the mini midline catheter had an average indwelling duration of (12.44 ± 6.66) days, with approximately 65% of patients retaining the catheter for over 1 week. This demonstrates its ability to adequately meet the 1–2 week treatment cycle requirements common in neurology, showing superior retention duration compared to a study involving emergency department patients (23). In-depth analysis of indwelling duration revealed two core influencing factors: treatment requirements and treatment outcomes.

First, this study identified a significant positive correlation between indwelling duration and average daily infusion volume (r = 0.351, *P* < 0.001). This aligns with Qin et al.'s (5) perspective that treatment requirements are the fundamental driver determining the selection and usage cycle of vascular access devices. Patients with high infusion volumes often present with more severe conditions or complex treatment regimens, inherently requiring longer treatment cycles and greater reliance on stable venous access. Consequently, elevated daily infusion volumes did not lead to earlier catheter removal but were associated with prolonged catheter retention. This indirectly reflects the mini midline catheter's reliable support for patients with high treatment demands, enabling them to complete prescribed treatment courses. This finding suggests that for neurology patients anticipated to require substantial daily infusion volumes, the mini midline catheter represents a superior alternative to traditional peripheral indwelling needles (24).

Second, the reason for catheter removal emerged as another significant factor influencing indwelling duration (*P* = 0.008). The vast majority (81.6%) of patients had their catheters removed due to successful completion of treatment, with their average dwell time constituting the bulk of this study's findings. This conclusion aligns with observations by Weber et al. (22) in medically hospitalized patients, where planned removal represents the most common outcome for midline catheters and best reflects their “on-demand placement” design intent. In contrast, unplanned catheter removal due to complications such as occlusion and leakage, though less frequent (11.8%), inevitably shortens the theoretical lifespan of the catheter (20), highlighting the importance of meticulous management in prolonging catheter dwell time. Furthermore, factors such as the choice of puncture vein, patient age, and target vein diameter showed no significant correlation with dwell time in this study. This differs slightly from some studies emphasizing the decisive role of vascular conditions. The findings may benefit from ultrasound guidance overcoming individual vascular variations and standardized catheterization and maintenance procedures, thereby mitigating the direct impact of these baseline factors on catheter durability (25, 26).

The analysis of catheter dwell time in this study holds clear clinical significance. First, it confirms that mini-midline catheters effectively accommodate the treatment needs of neurology patients, ranging from days to weeks, particularly for those undergoing intensive, well-defined treatment cycles. Second, it underscores that successful catheterization results from the combined effects of “appropriate selection” and “meticulous maintenance.” Future clinical practice can leverage these findings to: Precisely assess patients' treatment cycles and infusion volumes prior to catheter placement to select appropriate catheters; Maximize the utility of each catheter during indwelling through proactive, complication-preventive care, thereby optimizing both healthcare resource efficiency and patient safety.

## 5 Strengths and limitations

This study is a single-arm, prospective observational study without a control group. As this technique is a newly introduced procedure in our department, limitations may exist, such as insufficient operational experience and a limited number of cases. The use of convenience sampling may introduce selection bias, as the enrolled patients may not fully represent the entire neurology patient population. Furthermore, due to the limited number of complication events, the absence of multivariate regression analysis means that potential confounding variables cannot be statistically controlled; therefore, the identified associations should be considered exploratory rather than confirmatory. Conclusions should be interpreted with caution. Our findings primarily support the feasibility and safety of this technique; however, its comparative efficacy cannot be determined from this single-arm design. Future research requires multicenter, large-sample randomized controlled trials that directly compare this method with conventional peripheral venous access to further validate its overall benefits and provide a higher level of evidence-based support for clinical practice.

## 6 Conclusion

In summary, the mini midline catheter placed using ultrasound guidance combined with the accelerated Seldinger technique appears to be a feasible and potentially safe vascular access option for neurology patients requiring short-to-medium-term venous therapy, particularly those with significant fluid requirements. In this observational cohort, this approach was associated with high catheter placement success rates, low complication incidence, and high patient satisfaction. Randomized controlled trials are necessary to confirm these findings and establish its comparative effectiveness.

## Data Availability

The raw data supporting the conclusions of this article will be made available by the authors, without undue reservation.
